# Renal Epithelioid Angiomyolipoma in Children

**DOI:** 10.15586/jkcvhl.v8i2.178

**Published:** 2021-06-04

**Authors:** Dhruv Mahajan, Vishesh Jain, Sandeep Agarwala, Manisha Jana, Prashant P Ramteke

**Affiliations:** 1 Departments of Pediatric Surgery, All India Institute of Medical Sciences, Ansari Nagar, New Delhi, India;; 2 Departments of Radiology, All India Institute of Medical Sciences, Ansari Nagar, New Delhi, India;; 3 Departments of Pathology, All India Institute of Medical Sciences, Ansari Nagar, New Delhi, India

**Keywords:** angiomyolipoma, kidney tumor, pediatric cancer, renal epithelioid angiomyolipoma, tuberous sclerosis

## Abstract

Renal angiomyolipoma is a rare cause of renal tumor in children. Most are associated with tuberous sclerosis, and the classic type is observed more commonly. Epithelioid angiomyolipoma is even rarer with only limited case reports and series published in literature, most of which are of adult patients. We describe a 12-year-old boy, a diagnosed patient of tuberous sclerosis, who presented with pain in the left flank. On evaluation, it was found to have a left renal mass with the clinical picture suggestive of renal cell carcinoma. Partial nephrectomy was performed and histopathology revealed epithelioid angiomyolipoma. The child was asymptomatic at follow-up after 3 months. Only a few such cases in children are found in literature, which are discussed alongside. Differential diagnosis of this rare tumor must be kept in mind in a renal tumor as surgery is generally curative in this possibly malignant tumor. Metastasis confers a poor prognosis. Chemotherapy is generally not effective, although various regimens have been tried. Tumor recurrence must be kept in mind and a follow-up after apparent complete remission is of paramount importance.

## Introduction

Renal epithelioid angiomyolipoma (EAML), described much more frequently in adults, is an extremely rare tumor in pediatrics. Most cases are associated with tuberous sclerosis. There is a definite malignant potential inherently associated with EAML, evident from published reports of distant metastasis in rare cases (1, 2). This is in contrast to the reasonably benign course of classic angiomyolipoma (AML). Resection is invariably required, with prognosis greatly dependent on the presence of metastasis. A case of EAML in a child with tuberous sclerosis is described in which partial nephrectomy was curative with a good outcome. As per available adult literature, one-third of cases show advanced disease necessitating expeditious treatment and close follow-up, not dissimilar to renal cell carcinoma (RCC) (3).

## Case Report

**Figure 1:**
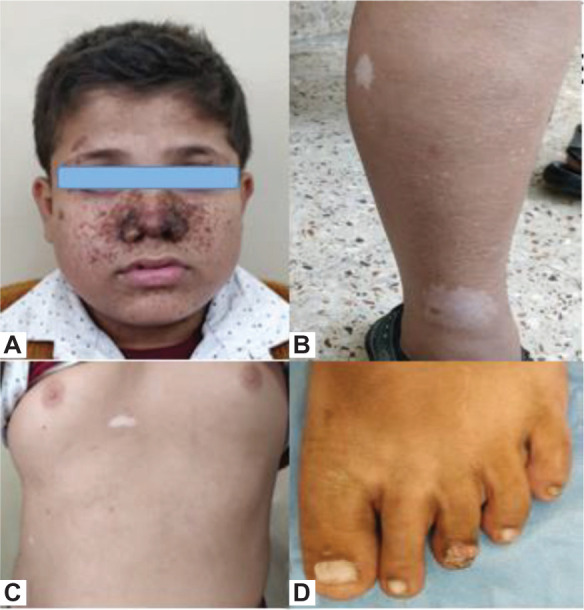
External clinical manifestations of tuberous sclerosis in the patient. (A) Facial angiofibromas in a classic butterfly pattern, adenoma sebaceum. Also seen is a typical forehead fibrous plaque, often seen as an early sign of tuberous sclerosis. (B) Multiple hypomelanic macules on the right lower limb, also known as ash leaf spots, caused due to lack of melanin. (C) Similar hypomelanic macule on the anterior chest wall. (D) Ungual fibroma/Koenen’s tumor present on the left 3rd toe.

A 12-year-old male child presented with episodic mild-to-moderate pain in the left flank for 1 month. There was no associated abdominal distension, lump, hematuria, fever, vomiting, or any other urinary complaints. There was no past history of any surgery. The child was diagnosed with tuberous sclerosis at the age of 8 years, when he presented with multiple seizures and skin manifestations characteristic of tuberous sclerosis. Previous abdominal ultrasonography performed at the age of 11 years was unremarkable. The child was well built with a visible shagreen patch, ash leaf macules, periungual fibromas, and hypomelanotic patches (Figure 1). On abdominal examination, a vague lump was palpable in the left lumbar and left hypochondrium with fullness of left renal angle. There was no history of tuberous sclerosis, malignancy, or renal disorder in any of the family members. Germline testing was not conducted.

**Figure 2:**
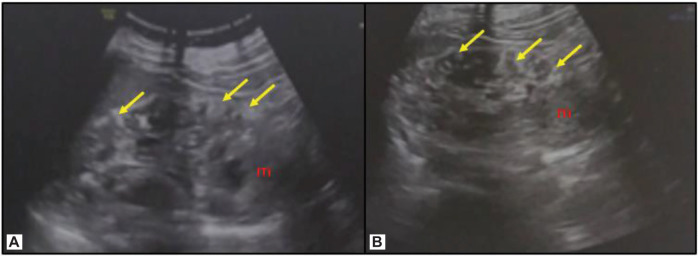
(A, B) Ultrasound images of the left kidney reveal multiple elongated echogenic focal lesions close to the cortex, suggesting angiomyolipomas. Note the mass from the lower pole (m).

**Figure 3:**
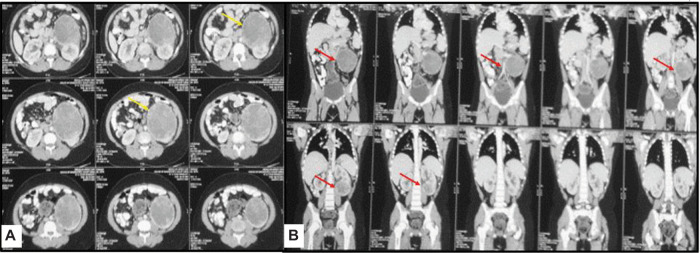
Images of (A) axial and (B) coronal CECT of the abdomen reveal exophytic heterogeneously enhancing solid mass (arrow) arising from the left kidney. The mass has no fat component in it.

An abdominal ultrasound revealed multiple well-defined elongated echogenic focal lesions close to the cortex in the right and left kidney, suggesting multiple angiomyolipomas, largest being 1.4 × 1.2 cm in size and a separate mass arising from the lower pole of the left kidney (Figure 2). A contrast-enhanced computed tomography (CECT) of the chest and abdomen was performed, which revealed bilateral, multiple, small, non-calcified, and enhancing cortical lesions containing fat that was suggestive of renal angiomyolipomas. Alongside, a 11 × 10 × 9 cm heterogeneously enhancing exophytic solid mass was seen arising from the lower pole of the left kidney causing mild left ureteric compression and mild hydroureteronephrosis (Figure 3). The mass had no fat component. A provisional diagnosis of left RCC was kept, with the rare possibility of epithelioid angiomyolipoma due to the rarity of this tumor.

On laparotomy, there was a tumor arising from the lower pole of the left kidney reaching up to the pelvis. The ureter, renal vessels, inferior vena cava, and surrounding organs were free of tumor. Left partial nephrectomy was performed, taking rim of normal surrounding renal tissue. The patient was discharged in stable condition with a normal postoperative course.

Overall, it was a tan brown, friable, well-circumscribed, and encapsulated tumor measuring 9 × 8.5 × 5 cm. On microscopic examination, the tumor was examined as predominantly composed of epithelioid (polygonal) cells, with focal areas showing thick-walled blood vessels and interspersed fat cells.

**Figure 4:**
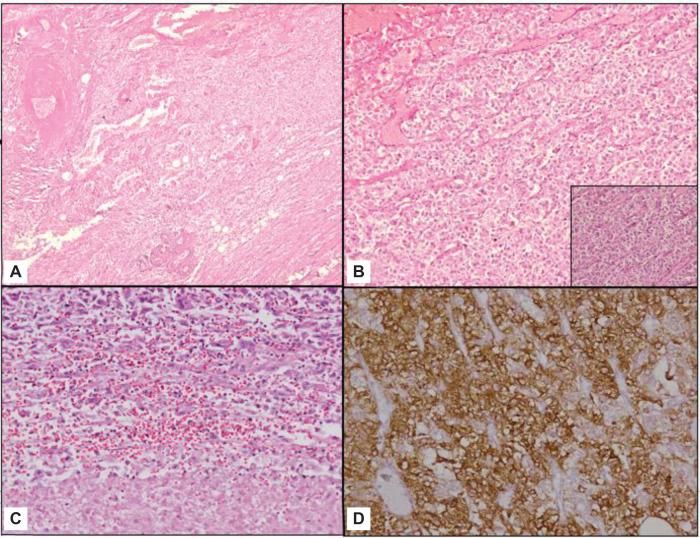
Histopathological findings of epithelioid angiomyolipoma. (A) Section from the mass shows a tumor predominantly composed of polygonal cells with focally present thick vessels and interspersed fat cells (hematoxylin and eosin × 40). (B) The tumor cells are predominantly epithelioid (polygonal), arranged in a nesting pattern (hematoxylin and eosin × 100). Inset comprised a high magnification image of tumor where tumor cells show moderate to abundant and clear to granular eosinophilic cytoplasm with distinct cytoplasmic boundries (hematoxylin and eosin × 200). (C) The tumor also shows large areas of necrosis. The surrounding tumor cells show moderate nuclear atypia and prominent nucleoli (hematoxylin and eosin × 200). (D) Tumor cells are immunopositive for HMB45 (× 200).

There were large areas of necrosis and perinecrotic tumor cells pointing moderate nuclear atypia and prominent nucleoli. There were satellite nodules in adjoining renal parenchyma. On immunohistochemistry, the tumor cells were immunopositive for HMB45, Melan A, and S100 (Figure 4). Based on the above-mentioned histomorphological and immunohistochemical features, it was diagnosed as epithelioid angiomyolipoma. The tumor was 0.1 cm away from the renal capsule, <0.1 cm from the resected margin, 0.2 cm from perirenal fat. Genomic testing of the tumor tissue was not performed.

Evaluation of the patient on follow-up at 3 months was unremarkable.

## Discussion

Renal angiomyolipoma is an uncommon cause of renal mass in children, which is classified as of either benign triphasic type (including classic AML and fat-poor AML) or potentially malignant epithelioid AML. As the name suggests, AML comprises a mixture of mature adipose tissue, smooth muscle cells, and blood vessels. AML is considered to be a benign clonal mesenchymal neoplasm (4), which is mainly renal in origin, albeit rarely arising from the liver. Both sporadic and syndromic types have been described, most commonly associated with the tuberous sclerosis complex (TSC), which is a group of autosomal dominant genetic disorders caused by germline mutations in *TSC1* or *TSC2* genes (5). Proteins hamartin and tuberin are encoded by *TSC1* and *TSC2* genes, respectively.

Recently, it has been further classified under a family of related mesenchymal neoplasms named “PEComas,” which includes angiomyolipoma, an entity called clear cell sugar tumor (CCSK) of the lung, lymphangiomyomatosis, and a group of rare, morphologically, and immunophenotypically similar lesions arising at a variety of visceral and soft tissue sites (6). This classification is based on the presence of a characteristic cell type—the perivascular epithelioid cell (PEC) in all these neoplasms. This further convolutes the pathological diagnosis of this entity concerning whether it is hamartoma, choristoma, or true neoplasm (7). No definitive etiological basis has been described in its pathogenesis.

A literature search to the best of our knowledge brought to light roughly 200 odd cases of EAML in adults and six cases in the pediatric population aged less than 18 years. The most common clinical presentation in the pediatric population is an abdominal lump, followed by abdominal pain. Associated history of tuberous sclerosis in a patient with renal lump vehemently insinuates the possibility of AML. Abdominal ultrasonography and CECT reveal renal mass with few definitive differentiating factors. The dearth of macroscopic fat in CT is a classic feature that differentiates classic AML from EAML, although infrequently macroscopic fat may not be visible even in classic AML. Calcifications, if seen, point more toward a diagnosis of RCC. Recently, chemical shift magnetic resonance imaging (MRI) has been reported for distinguishing minimal-fat renal AML from RCC (8), although it has not been studied in EAML. Dual-tracer positron emission tomography (PET)/CT has been also found use in differentiating fat-poor AML from RCC (7). However, in most instances, as in our patient; definitive distinguishing features between EAML and RCC are seldom present in CT. Thus, early prognostication remains an arduous job, or rather implausible, since EAML might have malignant potential and RCC has a poorer outcome than the relatively benign classic AML.

The presence of malignant potential in EAML has been attributed to the presence of tuberous sclerosis syndrome, extra-renal extension or renal vein involvement, tumor necrosis, carcinoma-type histology, and a tumor size of more than 7 cm (9). Only two cases of malignant EAML have been described in the pediatric population, one of which had the presence of tuberous sclerosis syndrome and another had extra-renal extension (1, 2). Presence of nuclear atypia or atypical mitosis could be the pointers for a possibility of an aggressively behaving tumor.

Surgery is the treatment of choice and is curative in most cases. Imaging is seldom able to convincingly rule out RCC, especially in an older children. Hence, resection is both diagnostic and therapeutic. Presence of metastasis dampens the survival rate. Various chemotherapeutic regimens have been tried in malignant EAML, without much success and the prognosis remains dismal in such cases. Tuberous sclerosis is caused by a decreased or absent expression of hamartin or tuberin. The hamartin–tuberin complex normally inhibits mammalian target of rapamycin complex 1 (mTORC1) in a cell. The loss of this complex in tuberous sclerosis leads to activation of constitutive mTORC1, leading to aberrant cellular signaling, and thus heading to tumor growth such as angiomyolipoma. Everolimus is a rapamycin derivative that inhibits the mTOR pathway by acting on mTORC1. In a randomized trial, everolimus was found to reduce volume of angiomyolipoma with an acceptable safety profile (10). Sirolimus, another mTOR inhibitor, has been found to have a role in angiomyolipoma, subependymal giant-cell astrocytoma, and lymphangioleiomyomatosis associated with tuberous sclerosis (11). These drugs could play a major role in the prospective future management protocols for tuberous sclerosis-associated angiomyolipoma.

Nephron sparing surgery has a definite role, as the goal must always be renal preservation as these patients might also have associated other angiomyolipomas. As in our patient, partial nephrectomy was generally possible, and must be strived for. Nese et al. established recurrence after complete resection in five of 33 adult patients at a mean period of 32 months (9). Similarly, recurrence is seen in two of six pediatric patients as described in Table 1. This necessitates a vigorous follow-up even after curative surgery and complete remission.

**Table 1:** Demography, clinical presentation, evaluation, management, and outcome of EAML in children aged less than 18 years described in literature.

S. No.	Publication	Age/sex	Presenting symptoms	Association with TSC	Family history	Computed tomography findings	Metastasis	Surgery	Adjuvant therapy	Histopathology	IHC markers	Complications/follow-up
1.	Gupta et al. (12)	4 years/female	Right abdominal mass	Present	None	Large contrast-enhancing mass in the right kidney replacing normal parenchyma	Absent	Right nephroureterectomy with lymph node sampling	None	Predominantly polygonal epithelioid cells with distinct cellular outlines and abundant granular eosinophilic cytoplasm with interspersed dysmorphic thick-walled blood vessels. No nuclear pleomorphism	HMB-45 + SMA + CK – EMA – Desmin – Myogenin – CD 117 – WT1 –	None/21 months, stable
2.	Johnson et al. (13)	17 years/female	Left renal mass	Present	Not mentioned	Multiple bilateral angiomyolipomas, all less than 1 cm, a new 5 cm exophytic enhancing fat poor solid mass	Absent	Left robotic-assisted laparoscopic partial nephrectomy	None	Predominantly polygonal epithelioid cells with abundant eosinophilic cytoplasm, mild nuclear atypia, and absence of mitotic activity	Vimentin + Actin + (limited) MART/melan-A + (diffused)	None
3.	Xi et al. (2)	7 years/male	Abdominal distension and pain	Absent	None	Large heterogeneous upper pole mass of the right kidney measuring 15 × 12 × 8 cm with tumor thrombus in the ipsilateral renal vein extending into the vena cava	Present	Partial tumor resection	7 courses of CAV/EP chemotherapy, temozolomide chemotherapy, partial tumor resection, temozolomide chemotherapy, thrombectomy, chemotherapy	Polygonalepithelioid cells, with eosinophilic or slightly eosinophilic cytoplasm and nuclear pleomorphism. Thick-walled blood vessels also seen. Calcification and hemorrhage also observed.	HMB-45 + Melan-A + ^d^SMA + S-100 + ^e^EMA – ^e^CK – Vimentin – Desmin – Chromogranin A – Synaptophysin – TFE3 –	Tumor metastasized to the liver and later to the bilateral lung, patient expired
4.	Citak et al. (1)	12 years/male	Abdominal pain and left abdominal mass	Present	Not mentioned	Giant mass originating from the left kidney	Present	Radical nephrectomy	Received everolimus. Had almost complete remission. Had progression of tumor, debulking surgery performed, axitinib treatment	Malignant EAML with nuclear pleomorphism, necrosis, and atypical mitotic figures	Cathepsin K + Melan-A + HMB45 + (focal) EMA + PAX8 + Cam5.2 + (rare cells)	Metastasis of the lung, progression of tumor, patient expired
5.	Kakaje et al. (14)	30 months/male	Dry cough, respiratory difficulty, incidentally detected abdominal lump	Absent	Not mentioned	Nodular mass surrounding the aorta and vena cava, anterior to the vertebrae, extending from the left kidney to right kidney. Chest Chylothorax	Absent	Right radical nephrectomy	None	Giant, mature polygonal epithelioid cells admixed with smooth muscle cells, scarce fat cells and vessels. No nuclear pleomorphism or atypical mitosis	-	None/6 months, stable
6.	Index case	12 years/male	Left abdominal pain	Present	None	Mildly bulky, solid cystic lesion with subcapsular spread, arising from lower pole of the left kidney	Absent	Left partial nephrectomy	None	Features of epithelioid angiomyolipoma with large areas of necrosis with a satellite nodule in adjoining renal parenchyma	HMB45 + Melan A + S100 +	None/1 month, stable

TSC: tuberous sclerosis, IHC: immunohistochemistry, HMB45: human melanoma black 45, SMA: smooth muscle actin, CK: cytokeratin, EMA: epithelial membrane antigen, CAV/EP: cyclophosphamide, doxorubicin, vincristine/etoposide, cisplatin.

Since very few pediatric cases are reported, it is difficult to predict relapse rate in this group. According to adult literature, proportions of relapse and metastasis are 17% and 49%, respectively (9). The median time of metastasis during follow-up is 12–18 months (1). Thus, a diligent follow-up with close monitoring is of paramount importance, considering the known potential for recurrence and metastasis.

A 3-month follow-up with clinical evaluation and relevant radiological investigations for 2 years is imperative for EAML. An abdominal ultrasound alternated with a CT scan seems a reasonable option to ensure a good yield of picking up any relapse. Biannual follow-up up to 5 years must be undertaken followed by annual follow-up as is the protocol for most tuberous sclerosis patients.

## Conclusion

EAML is seen sporadically in children, but in patients diagnosed with tuberous sclerosis, this must be kept in the differential. Conventional imaging is rarely conclusive. Use of chemical shift MRI and dual-tracer PET/CT is considered, but evidence-based validation is lacking. Surgery is the treatment of choice, with the goal being nephron-sparing surgery. Stringent follow-up is imperative as recurrence is a possibility.
